# Video killed the multiple-choice quiz: capturing pharmacy students' literature searching skills using a screencast video assignment

**DOI:** 10.5195/jmla.2021.1270

**Published:** 2021-10-01

**Authors:** Emily P. Jones, Christopher S. Wisniewski

**Affiliations:** 1 epjones3@email.unc.edu, Health Sciences Librarian, Health Sciences Library, University of North Carolina at Chapel Hill, Chapel Hill, NC; 2 wisniews@musc.edu, Professor, Department of Clinical Pharmacy and Outcome Sciences, Medical University of South Carolina, Charleston, SC

**Keywords:** screencast videos, competency-based assignment, pharmacy students, drug information

## Abstract

**Background:**

In a flipped, required first-year drug information course, students were taught the systematic approach to answering drug information questions, commonly utilized resources, and literature searching. As co-coordinator, a librarian taught three weeks of the course focused on mobile applications, development of literature searching skills, and practicing in PubMed. Course assignments were redesigned in 2019 based on assessment best practices and replaced weekly multiple-choice quizzes used in prior iterations of the course.

**Case Presentation:**

Following two weeks of literature searching instruction, students were assigned a drug information question that would serve as the impetus for the search they conducted. Students (n=66) had one week to practice and record a screencast video of their search in PubMed. Students narrated their video with an explanation of the actions being performed and were assessed using a twenty-point rubric created by the course coordinator and librarian. The librarian also created general feedback videos for each question by recording screencasts while performing the literature searches and clarifying troublesome aspects for students. The librarian spent about twenty-four hours grading and six hours writing scripts, recording, and editing feedback videos.

**Conclusion:**

Most students performed well on the assignment and few experienced technical difficulties. Instructors will use this assignment and feedback method in the future. Screencast videos proved an innovative way to assess student knowledge and to provide feedback on literature searching assignments. This method is transferrable to any medical education setting and could be used across all health professions to improve information literacy skills.

## BACKGROUND

Adult learning theory and instructional design best practices have long established that effective assessment techniques are linked to desired learning outcomes [1–7]. Additionally, assessments should be constructed in a manner that can actually measure student learning of material. Backward design is a particularly effective instructional design technique in which one begins with the end goal first and then works backward to develop appropriate assessment methodologies, learning activities, and course content [8]. This approach helps ensure planned learning activities and assessments align with desired learning outcomes. Despite helpful frameworks and learning theory as guidance, multiple-choice question assessments are often used in higher education, particularly in medical education, even when other methodologies are better suited to test skill acquisition or competencies [9]. As such, real-life examples of how educational activities are developed using backward design may prove helpful to educators who can utilize them to create assignments with this technique. This report presents one example of how librarians can move beyond multiple-choice question assessments and develop and implement innovative assignments to better evaluate student learning.

The educational activity was first implemented in Introduction to Drug Information, a course required in the first year of a traditional four-year Doctor of Pharmacy curriculum at the Medical University of South Carolina in fall 2019. The focus of this course was to teach the systematic approach to answering drug information questions [10] and to simulate real-world experiences for students to practice utilizing various tertiary and secondary resources. Course content was divided into four main components: identifying genuine need and categorization (i.e., designating questions to predefined drug information categories [11] like adverse effects, drug interactions, and pregnancy/lactation), tertiary resources, secondary resources, and analysis and synthesis.

The course was delivered via a flipped format where students watched instructor-created videos and engaged with material before attending a live class session focused on active learning and practical application of previously taught content. Student grades were comprised of six different components (Table 1), and final grades were reported on a pass-fail grading scale; a passing grade was determined by an overall course score of 70% or greater. The librarian was responsible for teaching approximately one-third of course content (specifically, weeks 6, 8, and 9) pertaining to mobile applications, secondary databases, and literature searching (Table 1).

**Table 1:** Course schedule

Systematic approach component	Weekly schedule
Identifying genuine need and categorization	Week 1—Background & ultimate question development & categorization
	Week 2—Systematic approach quiz due*
Tertiary	Week 3—Tertiary resources characteristics & utilization
	Week 4—Online tertiary compendia
	Week 5—Internet resources
	Week 6—Mobile applications
	Week 7—Tertiary resources quiz due*
Secondary	Week 8—Literature searching basics
	Week 9—Advanced literature searching techniques & practice
	Week 10—Literature searching video due*
Analysis and synthesis	Week 11—Question #1: Drug information center case
	Week 12—Question #2: Community case
	Week 13—Question #3: Hospital case
	Week 14—Question #4: Clinical case
	Week 15—Drug information question response due*

* With the exception of the drug information question response (50%), all assignments listed, plus participation and attendance, were each worth 10%

The librarian and course coordinator revised the summative assessment of literature searching ability from multiple-choice quizzes used in previous years to a skills-based competency. This change was made due to personal reflection of how to better assess student knowledge of literature searching skills using a backward design approach. The objective of this report is to describe the development of this innovative assessment methodology and to provide information regarding student performance and feedback on the competency-based assignment.

## CASE PRESENTATION

During two weeks of instruction covering literature searching using PubMed, students were taught a broad range of topics. Concepts included controlled vocabulary and text terms, Boolean operators, expanding or narrowing search results, and phrase searching and truncation. As an introductory course, the goal of these instruction sessions was to provide students with a broad overview of literature searching mechanics that they could refine toward expertise during their education.

Following introduction of these concepts, students were assigned a drug information question that served as the impetus for the literature searching assignment. Eight total questions, two each related to a specific category, were divided among the students. The categories and an example question from each is presented in Table 2.

**Table 2:** Drug information categories and questions used in assignment

Category	Question
Compounding	Does cinacalcet come as a suspension? If not, can you make it?
Drug-lab interaction	What drugs can cause a false positive benzodiazepine result on a urine drug screen?
Herbal product	Can kava be used to treat insomnia?
Method of administration	Can warfarin be given through a feeding tube?

A twenty-point rubric for this assignment was created to encourage students to develop, but not master, these skills (Appendix A). The rubric was peer reviewed by two other librarians for clarity. The rubric was then provided to students in the assignment instructions and integrated into the learning management system (LMS) gradebook.

Students (n=66) had one week to practice and record a screencast video of their finalized literature search in PubMed utilizing an institutional subscription to Panopto, an online video platform. Assignment instructions (Appendix B) indicated the video should be five minutes or less and that students should narrate their video with an explanation of the actions being performed. Students were provided the grading rubric prior to completing the assignment.

All students completed the literature searching video assignment. Overall, students performed well on the assignment [mean score=17.35/20; 86.75% (IQR 80–90)]. Few experienced technical difficulties, and those who did were given additional information on how to record or upload their videos.

The librarian also created general feedback videos for each drug information question (eight total) utilizing Panopto. Videos depicted the librarian performing searches while following the rubric and clarifying troublesome aspects for students. Feedback videos were available to students via the LMS after grades were released. In total, the librarian spent about twenty-four hours grading and six hours writing scripts, recording, and editing feedback videos.

## DISCUSSION

Overall, this assignment was well received by students; 91% of students stated they felt their literature searching skills improved in a course evaluation survey, a notable improvement from 83% the prior year. Instructors anticipated there to be more technical issues with completing the assignment. To the best of the authors' knowledge, this is the first assignment in the curriculum that required students to record a screencast video. Despite their lack of exposure to this technique, students required minimal instruction on how to create their screencast video using Panopto or how to upload it to the LMS; these instructions were provided in writing in the assignment handout (Appendix B). Few issues were encountered and were easily circumvented.

While librarian-created screencasts to teach database searching are well established in the literature, particularly in the form of tutorial videos [12–20], literature on librarian use of screencast assignments to assess student learning is sparse. The only publication identified is by Kuban and Mulligan [21], who wrote about a database searching screencast assignment in a journalism research course taught mainly to first-year undergraduates. The focus of their assignment was to teach introductory information literacy skills. Their rubric consisted of five elements, only one of which was specific to database searching mechanics. The other elements focused on background information of the database, when and why to search the specified database, exporting results, and credibility of an identified source.

To the best of the authors' knowledge, there are no publications on student-created screencasts as literature searching assignments in any health professions educational setting. Therefore, this screencast method of assessment is innovative and novel in health care education and can easily be used to evaluate literature searching abilities of students irrespective of discipline. While implementation of an assignment as this may have existed in the past, the ability to create this type of assignment is readily available to students via freely available software, institutionally subscribed resources, or their personal smartphone or tablet. Screencast assignments are documented in the literature of other disciplines like accounting [22–24] and education [25, 26]. Faculty use of screencasts to deliver feedback on assignments is also demonstrated in the literature and was perceived by students taking part in the published studies to be more effective and favorable to written feedback [27–30].

Utilizing this skills-based competency instead of multiple-choice quizzes did add a moderate time strain on the librarian. The amount of time dedicated to this assignment was mostly devoted to grading student videos. This was the first year a competency-based assignment was implemented, and it is possible grading will take less time in future iterations as the librarian becomes more familiar with the process.

Instructors felt implementing this original and modern assignment was well worth the time investment compared to multiple-choice quizzes used in previous iterations of the course for multiple reasons. First, while students were instructed to work individually on multiple-choice quizzes used in prior years, there is no guarantee they did so. The screencast assignment ensured each student completed the assignment individually and provided more useful information to instructors about student learning. The videos allowed instructors to see exactly where students struggled in both the actions being performed or the narration of the video. Alternatively, student performance on multiple-choice quizzes can be misleading; students can correctly guess on questions to which they do not actually know the answer, thus providing inaccurate feedback about student knowledge. This screencast assignment also ensured student knowledge of literature searching would be assessed based on their ability to perform a literature search, not rote memorization or guesswork.

Writing scripts for feedback videos and then recording and editing them also required significant time. The librarian was very familiar with the process of writing scripts and recording screencasts using Panopto and had performed these tasks for numerous videos as part of the flipped classroom design of this course in the three years prior to this assignment. It was important to the instructors to provide clear guidance on this assignment to students, and both educators felt it would be more effective to visually see and hear explanations behind those actions instead of written feedback. Additionally, it was important to provide useful feedback because the final assignment, a written drug information question response, applied to the same case used for the literature searching assignment. Designed based on scaffolding learning theory [31], students continued with the assigned drug information question and had to utilize skills learned throughout the entire semester to find applicable information and provide a written response.

Instructors noted sections of the rubric that students expressed to be unclear after grades were released. For example, students thought they should get full credit for showing use of Boolean operators in the search, not understanding they needed to use them optimally to receive full points. In addition to clarifying how advanced searching is defined, incentive for keeping the video under five minutes was added by removing one point from the credit for article submission. The initial rubric used in fall 2019 can be found in Appendix A and the revised version, used in fall 2020 and after, in Appendix B.

In conclusion, screencast videos proved an innovative way to assess student knowledge and to provide feedback on literature searching assignments. Instructors feel this competency-based assignment was a better gauge of student knowledge than multiple-choice quizzes. Additionally, students received individualized, written feedback via the rubric and general feedback, presented visually and auditorily, via librarian-created screencasts. Instructors feel delivery of feedback through screencasts could have been more meaningful to students because it was presented in the same manner students completed the assignment. Instructors will continue to use this method for assignment and feedback in the future, with minor changes to the rubric planned before the next course offering to decrease student confusion. This method of assessing literature searching skills can be easily extrapolated to the education of students training in other medical fields.

## Data Availability

There are no data associated with this article.

## SUPPLEMENTAL FILES

**Appendix A.** Literature Searching Video Assignment Fall 2019

**Appendix B.** Literature Searching Video Assignment Fall 2020
