# Supplementary material for: Video killed the multiple-choice quiz: capturing pharmacy students' literature searching skills using a screencast video assignment
Source: J Med Libr Assoc. 2021 Oct 1;109(4):672–6. doi: 10.5195/jmla.2021.1270 (PMC8608213; doi:10.5195/jmla.2021.1270)
Supplement: Supplementary file 1 — Appendix A. Literature Searching Video Assignment Fall 2019 [file jmla-109-4-672-s01.docx]

**Appendix A**

Literature Searching Video Assignment Fall 2019

| Literature Searching Video Grading Rubric | | | |
| --- | --- | --- | --- |
| Access: Does the student access PubMed® from the MUSC Libraries website? | | | |
| No (0 points) | | Yes (1 point) | |
| Narration: Does the student clearly explain the actions being performed in the video? | | | |
| No (0 points) | | Yes (2 points) | |
| MeSH Database: Does the student begin the literature search by searching the MeSH database? | | | |
| No (0 points) | | Yes (2 points) | |
| Appropriate MeSH Terms: Does the student select the appropriate MeSH terms for the case? | | | |
| None were appropriate (0 points) | Some were appropriate (2 points) | | All were appropriate (4 points) |
| Boolean Operators: Does the student combine MeSH terms with appropriate Boolean operators? | | | |
| No (0 points) | | Yes (1 point) | |
| Advanced Searching Techniques: Does the student use advanced techniques to identify search results? | | | |
| No (0 points) | | Yes (2 points) | |
| Search Results: Does the search yield an efficient number of appropriate results? | | | |
| Search yields largely inappropriate literature (0 points) | Inefficient search yields largely appropriate results (2 points) | | Efficient search yields largely appropriate results (4 points) |
| Non-Indexed Citations: After completing the MeSH search, does the student appropriately conduct a search that will retrieve non-indexed citations? | | | |
| No (0 points) | | Yes (2 points) | |
| Article Submission: Was the PDF the student submitted relevant and yielded from the recorded literature search? | | | |
| No (0 points) | | Yes (2 points) | |
