# Supplementary material for: Video killed the multiple-choice quiz: capturing pharmacy students' literature searching skills using a screencast video assignment
Source: J Med Libr Assoc. 2021 Oct 1;109(4):672–6. doi: 10.5195/jmla.2021.1270 (PMC8608213; doi:10.5195/jmla.2021.1270)
Supplement: Supplementary file 2 — Appendix B. Literature Searching Video Assignment Fall 2020 [file jmla-109-4-672-s02.docx]

Appendix B

Literature Searching Video Assignment Fall 2020

Learning Objectives**:**

- Create a screencast video while conducting a literature search in PubMed
- Utilize basic and advanced searching techniques
- Explain reasoning for choosing terms and the actions being performed

Overview**:** Using Panopto™, students will record a short video **(≤ 5 minutes**) of their computer screen while performing a literature search in PubMed. This search should help identify literature appropriate for the assigned drug information (DI) question case. Students are expected to use a microphone and narrate the steps they are taking as they perform the search that add reasoning behind those decisions (eg, what are the major concepts of the case; why was a specific MeSH term chosen over others; why are text words being added to the search). Students do not need to record their faces, only the screen itself. Students can earn up to 20 points via a rubric (found on page 3) for this assignment.

Logistics**:**

- Students will record their presentation using Panopto™ on their own time. [Recording instructions](https://support.panopto.com/s/topic/0TO39000000EovdGAC/create) are available online. Save the presentation with **the file titled as your name** to the [assignments] folder associated with the course.


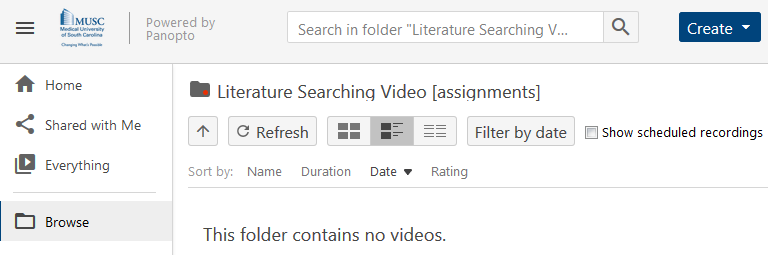


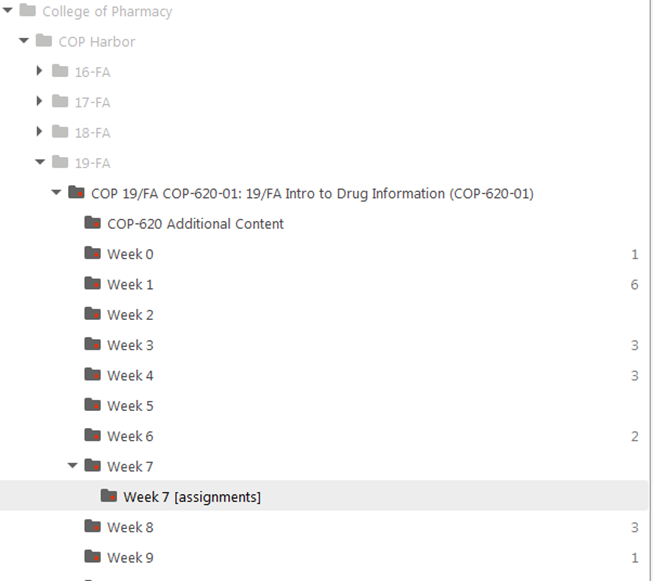

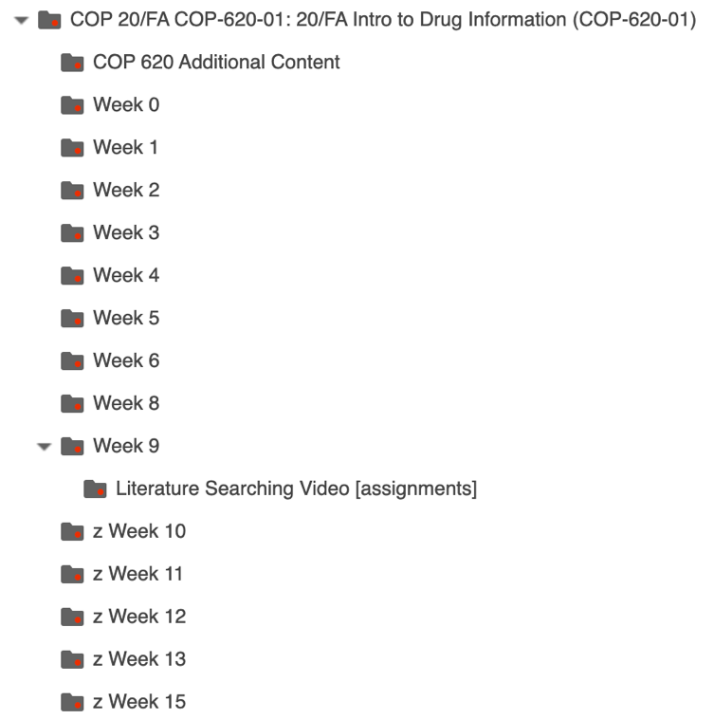


- Each student will submit a PDF of an article identified via the recorded literature search.
- By **11:59 PM on** **November 5, 2020**, the **PDF of the identified article** must be submitted to Moodle **and** **literature-searching videos** must be recorded and submitted to Panopto™. Failure to submit the article or record and submit the presentation by the deadline will result in a 0; therefore, plan to record the presentation well in advance of the deadline in case technological issues arise. Should problems arise, contact the course coordinator prior to the deadline.

# Tips for Success:

- Remember to use the systematic approach, categorizing the question and reviewing tertiary resources, for the reasons we have discussed, before searching.
- Take time conducting the search and look multiple ways before initiating any type of recording.
- While searching, document your search strategy so that when your search is the one you want to record and submit, all you must do is restart that search from the beginning.
- Consider doing a test video to make sure that both the audio and video are working before recording the video for submission.
- Feel free to have searches prepared in another tab or window in advance and explain that when recording to shorten the length of the video.
- The question in this case is the same one you will be assigned for the final DI question assignment. As such, conducting a good search now will help you when you start working on answering this question at the end of the semester.

# Literature Searching Video Rubric:

Total Points Possible: 20

| Literature Searching Video Grading Rubric | | | |
| --- | --- | --- | --- |
| Access: Does the student access PubMed from the MUSC Libraries website? | | | |
| No (0 points) | | Yes (1 point) | |
| Narration: Does the student clearly explain the actions being performed in the video? | | | |
| No (0 points) | | Yes (2 points) | |
| MeSH Database: Does the student begin the literature search by searching the MeSH database? | | | |
| No (0 points) | | Yes (2 points) | |
| Appropriate MeSH Terms: Does the student select the appropriate MeSH terms for the case? | | | |
| None were appropriate (0 points) | Some were appropriate (2 points) | | All were appropriate (4 points) |
| Boolean Operators: Does the student combine MeSH terms with appropriate Boolean operators? | | | |
| No (0 points) | | Yes (1 point) | |
| Advanced Searching Techniques: Does the student use advanced techniques (ie, not the advanced search builder) other than Boolean operators to identify search results? | | | |
| No (0 points) | | Yes (2 points) | |
| Search Results: Does the search yield an efficient number of appropriate results? | | | |
| Search yields largely inappropriate literature (0 points) | Inefficient search yields largely appropriate results (2 points) | | Efficient search yields largely appropriate results (4 points) |
| Non-Indexed Citations: After completing the MeSH search, does the student appropriately conduct a search that will retrieve non-indexed citations? | | | |
| No (0 points) | | Yes (2 points) | |
| Article Submission: Was the PDF the student submitted relevant and identified from the recorded literature search? | | | |
| No (0 points) | | Yes (1 point) | |
| Video Length: Was the video less than 5 minutes in length? | | | |
| No (0 points) | | Yes (1 point) | |
